# Mixed venous oxygen saturation identifies high-risk physiology in advanced cardiogenic shock

**DOI:** 10.62838/jccm-2026-0034

**Published:** 2026-10-30

**Authors:** Thang Xuan Pham, Minh Duy Nguyen, Hoi Van Dang, Dat Tien Nguyen, Thu Nguyen, Hung Tran Ha, Chi Van Nguyen, Quan Huu Nguyen, Tuan Anh Nguyen

**Affiliations:** Hanoi Medical University, Hanoi, Vietnam; Center for Emergency Medicine, Bach Mai Hospital, Hanoi, Vietnam

**Keywords:** Cardiogenic Shock, Hemodynamics, Pulmonary Artery Catheter, Microcirculation

## Abstract

**Background:**

Early identification and risk stratification in cardiogenic shock remain clinically important. Mixed venous oxygen saturation (SvO_2_) may provide additional information for identifying patients at higher risk within a given SCAI stage.

**Objectives:**

To evaluate the role of mixed venous oxygen saturation (SvO_2_) and hemodynamic parameters in combination with Society for Cardiovascular Angiography and Interventions (SCAI) staging, stratifying 30-day mortality risk in patients with cardiogenic shock due to acute myocardial infarction.

**Methods:**

A retrospective analysis of baseline data was conducted in 84 patients with cardiogenic shock due to acute myocardial infarction, selected from a larger study registered on ClinicalTrials.gov (NCT07062744). Hemodynamic parameters and indices of oxygen delivery–consumption were collected immediately after insertion of the pulmonary artery catheter (T0). The Society for Cardiovascular Angiography and Interventions (SCAI) stage was determined at the same time point. Associations between baseline hemodynamic variables and 30-day mortality were evaluated using group comparison tests, receiver operating characteristic (ROC) curve analysis, Kaplan–Meier survival analysis with log-rank testing, and multivariable Cox proportional hazards regression. The proportional hazards assumption was assessed by including time-dependent interaction terms between each covariate and follow-up time. The primary outcome was all-cause mortality within 30 days, prespecified in the registered protocol.

**Results:**

Thirty-day mortality in the overall study population was 32.1% (27/84). Within the same SCAI stage, SvO_2_ further stratified patients into groups with different observed mortality risks. Mortality risk classification using a threshold of 53% of SvO2. Lower SvO_2_ was associated with reduced 30-day survival and remained associated with mortality risk after adjustment in Cox regression analysis.

**Conclusions:**

Mixed venous oxygen saturation (SvO_2_) with a threshold of 53% was associated with differences in 30-day mortality risk among patients with cardiogenic shock within the same SCAI stage. It may provide additional risk stratification during early invasive hemodynamic assessment.

## Introduction

Early risk stratification of patients with cardiogenic shock due to acute myocardial infarction remains challenging. Although SCAI staging has been widely adopted, this system is primarily based on clinical presentation and does not fully assess early physiological hemodynamic derangements [1]. In cardiogenic shock, reduced cardiac output impairs systemic oxygen delivery (DO_2_), leading to a compensatory increase in tissue oxygen extraction (O_2_ER) and a marked reduction in mixed venous oxygen saturation (SvO_2_) [2,3]. Current consensus statements emphasize early hemodynamic assessment in patients with persistent or advanced cardiogenic shock. Pulmonary artery catheterization (Swan–Ganz) enables direct measurement of cardiac output, ventricular filling pressures, and indices of global oxygen transport, thereby supporting physiological assessment and therapeutic decision-making. However, previous studies of pulmonary artery catheter-derived variables have typically included heterogeneous populations with cardiogenic shock from multiple etiologies. In addition, the timing of catheter placement has often been undefined, and many cohorts included patients already receiving mechanical circulatory support, which may substantially alter hemodynamic measurements [4,5,6]. In this study, the authors aimed to characterize early hemodynamic physiology in severe cardiogenic shock due to acute myocardial infarction (SCAI stage D and E). Pulmonary artery catheterization was performed shortly after coronary angiography (within 6 hours). No patients had received mechanical circulatory support at baseline hemodynamic assessment (T0). At this time point, the doses of vasoactive and inotropic agents were recorded and the vasoactive–inotropic score (VIS) was calculated. We therefore examined whether SvO_2_ measured at this early stage could stratify 30-day mortality risk within the same SCAI stage.

## METHODS

### Study design and trial registration

This study analyzes baseline hemodynamic physiology (T0) from a registered, single-center, single-arm interventional clinical trial (ClinicalTrials.gov ID: NCT07062744).

### Population and timing

Adult patients aged ≥18 years with cardiogenic shock secondary to acute myocardial infarction [7], were consecutively enrolled according to the predefined inclusion and exclusion criteria of the study protocol (ClinicalTrials.gov ID: NCT07062744).

**Figure:**
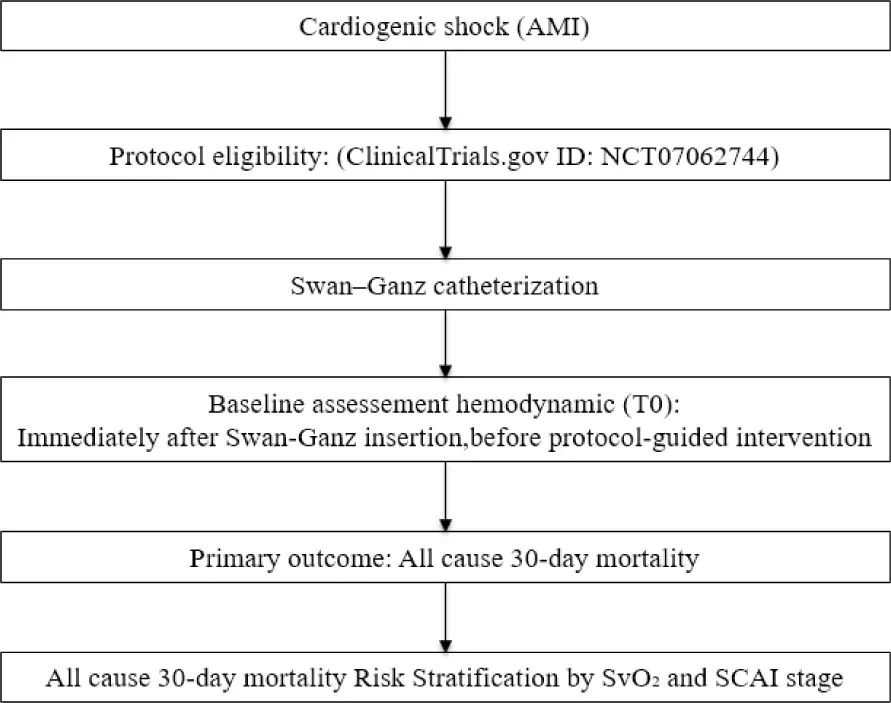


### Inclusion Criteria

- Age ≥ 18 years and voluntary consent.
- Diagnosed with cardiogenic shock based on IABP SHOCK II (2012) criteria:
  ○ SBP < 90 mmHg for ≥30 minutes or requiring vasopressors;
  ○ Evidence of end-organ damage with one of the following: Altered consciousness; Urine output < 30 ml/hour; Cold, mottled skin; Lactate > 2 mmol/L.
- SCAI stage D and E.
- Without mechanical circulatory support.
- Exclusion Criteria:
- Cellulitis at the catheter insertion site.
- Anatomic anomalies or prior radiation therapy to the neck.
- Coagulopathy (INR > 1.5 and/or platelet count < 50 G/L).
- End-stage chronic diseases (advanced cancer, late-stage HIV, bedbound >3 months, cirrhosis Child-Pugh C, dialysis-dependent CKD).
- Cardiac arrest or mechanical complications (e.g., ventricular wall rupture) before catheter insertion.
- Congenital heart disease or intracardiac shunts.
- Legal guardian or patient refusal.

### Hemodynamic monitoring and data collection

The pulmonary artery catheter (Swan–Ganz) was placed after coronary angiography and revascularization had been completed, and baseline hemodynamic measurements (T0) were obtained immediately after catheter insertion. These variables included cardiac index (CI), stroke volume index (SVI), cardiac power output (CPO), pulmonary artery occlusion pressure (PAOP), right atrial pressure (RAP), mixed venous oxygen saturation (SvO_2_), oxygen extraction ratio (O_2_ER), arterial lactate concentration, and the PCO_2_ gap/Ca–vO_2_ ratio. All measurements were obtained before protocol-driven adjustments of vasoactive agents, resuscitation fluids.

### Outcomes

30-day all-cause mortality was defined as the primary outcome.

### Statistical analysis

Statistical analyses were performed using SPSS software, version 20 (IBM Corp., Armonk, NY, USA). Data distribution was assessed using the Shapiro–Wilk test. Continuous variables are presented as mean ± standard deviation and were compared between groups using the Student's t-test or the Mann–Whitney U test, as appropriate according to data distribution. Categorical variables are expressed as counts and percentages and were compared using the χ^2^ test or Fisher's exact test. The primary endpoint was 30-day all-cause mortality. Time-to-event outcomes were analyzed using Kaplan–Meier survival curves and compared with the log-rank test. Receiver operating characteristic (ROC) curve analysis was used to evaluate the discriminative performance of baseline SvO_2_, vasoactive–inotropic score (VIS), and oxygen extraction ratio (O_2_ER). Optimal cut-off values were determined using the Youden index. Pairwise comparisons of AUCs were conducted using the DeLong method. All tests were two-sided, and a p-value < 0.05 was considered statistically significant.

### Ethical approval

The study protocol was reviewed and approved by the Institutional Ethics Committee (approval number: 33/BM-HĐĐĐ, dated May 21, 2025). All procedures were conducted in accordance with the Declaration of Helsinki. Written informed consent was obtained from all patients or their legally authorized representatives before study participation.

## Results

Baseline demographic and clinical characteristics of survivors and non-survivors are summarized in Table 1. There were no significant differences in age, body mass index, or length of ICU stay between survivors and non-survivors (all p > 0.05). AST and ALT levels were higher in non-survivors, although the differences did not reach statistical significance (AST: p = 0.062; ALT: p = 0.075). In contrast, total bilirubin was significantly higher in non-survivors compared with survivors (20.5 ± 20.4 vs. 11.6 ± 6.2 μmol/L, p = 0.033).

**Table 1:** Baseline Demographic and Clinical Characteristics of Survivors and Non-Survivors

**Variable**	**Survivors (n=57)**	**Non-survivors (n=27)**	**p value**
Age (years)	69.6 ± 16.9	70.9 ± 11.2	0.73^†^
BMI (kg/m^2^)	23.5 ± 3.7	23.5 ± 3.6	0.98^†^
Length of stay in ICU	6.05 ± 4.08	5.22 ± 3.48	0.34^††^
Creatinine (μmol/L)	190.5 ± 153.2	224.4 ± 186.1	0.42^††^
AST (U/L)	279.8 ± 397.6	756.8 ± 1244.5	0.062^††^
ALT (U/L)	135.3 ± 196.2	321.8 ± 507.8	0.075^††^
Total bilirubin (μmol/L)	11.6 ± 6.2	20.5 ± 20.4	0.033^††^
Hs-Troponin (ng/L)	3468 ± 3313	4842 ± 4035	0.13^††^
EF (%)	36.5 ± 10.5	32.9 ± 10.9	0.15^†^
SOFA score	9.9 ± 2.3	11.0 ± 1.9	0.023^†^
APACHE II score	19.3 ± 5.0	20.1 ± 5.4	0.52^†^

† p value calculated using the independent Student's t-test.

†† p value calculated using the Mann–Whitney U test.

**Abbreviations:** BMI, body mass index; ICU, intensive care unit; AST, aspartate aminotransferase; ALT, alanine aminotransferase; EF, left ventricular ejection fraction; SOFA, Sequential Organ Failure Assessment; APACHE II, Acute Physiology and Chronic Health Evaluation II.

Arterial, pulmonary artery–derived oxygenation, and perfusion variables are presented in Table 2. In terms of arterial oxygenation, PaO_2_ values were significantly lower among non-survivors compared with survivors (108 ± 40 vs. 144 ± 78 mmHg; p = 0.007). Pulmonary artery-derived indices of tissue oxygenation showed consistent differences according to out-come. Non-survivors demonstrated markedly lower PvO_2_ (31.6 ± 5.4 vs. 40.2 ± 11.3 mmHg; p < 0.001) and reduced mixed venous oxygen saturation (SvO_2_) compared with survivors (52.2 ± 13.0% vs. 62.6 ± 12.1%; p = 0.001). Analysis of oxygen transport variables revealed selective alterations in non-survivors. Mixed venous oxygen content (CvO_2_) was significantly lower in non-survivors (8.9 ± 3.1 vs. 10.9 ± 3.1 mL/dL; p = 0.009). Accordingly, the arteriovenous oxygen content difference [C(a–v)O_2_] was higher in non-survivors (7.2 ± 2.7 vs. 6.0 ± 2.4 mL/dL; p = 0.049), accompanied by an increased oxygen extraction ratio (42.6 ± 13.5% vs. 35.9 ± 12.0%; p = 0.032).

**Table 2:** Arterial, Pulmonary Artery–Derived Oxygenation, and Perfusion Parameters in Survivors and Non-Survivors

**Parameter (T0)**	**Survivors (n = 57)**	**Non-survivors (n = 27)**	**P value**
**Acid–base status (arterial)**			
pH	7.34 ± 0.10	7.32 ± 0.10	0.40^†^
HCO_3_^−^ (mmol/L)	22.4 ± 4.8	22.8 ± 6.7	0.74^†^
PaCO_2_ (mmHg)	37.9 ± 10.7	38.4 ± 9.3	0.82^†^
**Oxygenation (arterial)**			
PaO_2_ (mmHg)	144 ± 78	108 ± 40	0.007^†^
SaO_2_ (%)	97.8 ± 6.1	96.7 ± 4.6	0.36^†^
**Tissue oxygenation (PA)**			
PvO_2_ (mmHg)	40.2 ± 11.3	31.6 ± 5.4	<0.001^†^
SvO_2_ (%)	62.6 ± 12.1	52.2 ± 13.0	0.001^†^
**Lactat level**			
Lactate – arterial (mmol/L)	3.51 ± 2.49	3.58 ± 2.56	0.91^††^
Lactate – PA (mmol/L)	3.45 ± 2.42	3.52 ± 2.49	0.91^††^
**Oxygen transport and utilization parameters**			
CaO_2_ (mL/dL)	17.2 ± 3.5	16.4 ± 3.2	0.32^†^
CvO_2_ (mL/dL)	10.9 ± 3.1	8.9 ± 3.1	0.009^†^
C(a–v)O_2_ (mL/dL)	6.0 ± 2.4	7.2 ± 2.7	0.049^††^
O_2_ extraction ratio (%)	35.9 ± 12.0	42.6 ± 13.5	0.032^††^
DO_2_ (mL O_2_/min)	711.0 ± 370.9	603.5 ± 245.0	0.175^†^
VO_2_ (mL O_2_/min)	242.7 ± 134.2	240.5±89.8	0.939^†^
**Perfusion indices**			
PCO_2_ gap (mmHg)	7.6 ± 3.5	7.6 ± 4.2	0.99^†^
PCO_2_ gap / C(a–v)O_2_	1.57 ± 1.51	1.00 ± 0.68	0.019^††^

† p value calculated using the independent Student's t-test.

†† p value calculated using the Mann–Whitney U test.

Abbreviations: PaO_2_, arterial partial pressure of oxygen; SaO_2_, arterial oxygen saturation; PvO_2_, mixed venous partial pressure of oxygen; SvO_2_, mixed venous oxygen saturation; CaO_2_, arterial oxygen content; CvO_2_, venous oxygen content; C(a–v)O_2_, arteriovenous oxygen content difference; O_2_ER, oxygen extraction ratio; PCO_2_ gap, venous-to-arterial carbon dioxide partial pressure difference; PA, pulmonary artery.

Hemodynamic and oxygen transport variables are shown in Table 3. Stroke volume index was significantly lower among non-survivors (19.8 ± 4.9 vs. 23.4 ± 8.4 mL/m^2^; p = 0.018). Measures of cardiac power were likewise reduced in the non-survivor group, including cardiac power output (0.67 ± 0.25 vs. 0.82 ± 0.42 W; p = 0.041) and cardiac power index (0.41 ± 0.15 vs. 0.49 ± 0.23 W/m^2^; p = 0.047). Mixed venous oxygen saturation measured in the pulmonary artery was significantly lower in non-survivors than in survivors (56.5 ± 15.4% vs. 64.0 ± 11.5%; p = 0.014).

**Table 3:** Hemodynamic and Oxygen Transport Variables in Survivors and Non-Survivors

**Parameter Hemodynamics (T0)**	**Survivors (n=57)**	**Non-survivors (n=27)**	**P value**
Heart rate (bpm)	115.9 ± 21.2	119.5 ± 19.5	0.45^†^
MAP (mmHg)	83.1 ± 14.2	79.9 ± 16.1	0.38^†^
Cardiac index (L/min/m^2^)	2.47 ± 1.06	2.31 ± 0.63	0.37^†^
Stroke volume index (mL/m^2^)	23.4 ± 8.4	19.8 ± 4.9	0.018^†^
SVR index (dyn·s·cm^−5^·m^2^)	2688 ± 1114	2553 ± 1005	0.58^†^
Right atrial pressure (mmHg)	10.4 ± 5.3	11.4 ± 6.1	0.46^†^
Mean PAP (mmHg)	27.3 ± 8.6	29.8 ± 11.0	0.31^†^
PAPi	2.17 ± 2.27	2.88 ± 4.29	0.42^†^
PAOP (mmHg)	17.8 ± 7.2	20.3 ± 8.1	0.19^†^
Cardiac power output (W)	0.82 ± 0.42	0.67 ± 0.25	0.041^†^
Cardiac power index (W/m^2^)	0.49 ± 0.23	0.41 ± 0.15	0.047^†^
SvO_2_ (PAC, %)	64.0 ± 11.5	56.5 ± 15.4	0.014^†^
DO_2_ (mL/min)	694 ± 369	604 ± 197	0.15^††^
VO_2_ (mL/min)	232 ± 133	253 ± 95	0.42^††^
Vasoactive–inotropic score (VIS)	66 ± 68	135 ± 139	0.003^††^

† p value calculated using the independent Student's t-test.

†† p value calculated using the Mann–Whitney U test.

Abbreviations: MAP, mean arterial pressure; SVR, systemic vascular resistance; PAP, pulmonary artery pressure; PAPi, pulmonary artery pulsatility index; PAOP, pulmonary artery occlusion pressure; SvO_2_, mixed venous oxygen saturation; DO_2_, oxygen delivery; VO_2_, oxygen consumption; VIS, vasoactive–inotropic score.

ROC analysis (Figure 1) showed that SvO_2_ was associated with 30-day mortality (AUC 0.699; 95% CI 0.572–0.827; p = 0.002). A cutoff value of 53% was identified, with moderate sensitivity and specificity. VIS and O_2_ER also showed moderate discriminative ability. Comparisons between ROC curves using the DeLong test did not show statistically significant differences between the AUCs (all p > 0.05 with the De-Long test).

**Fig. 1.**
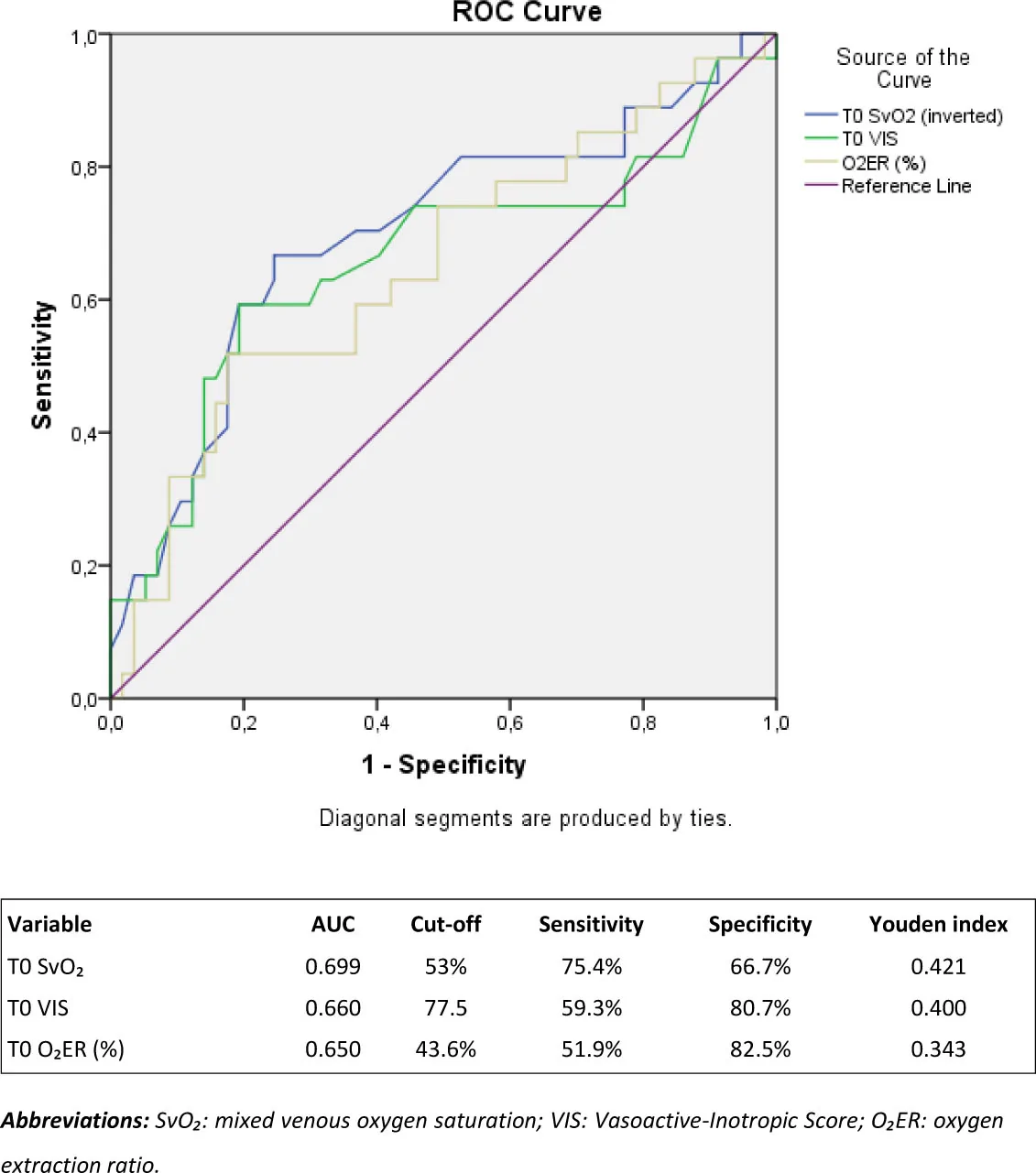
Receiver Operating Characteristic Analysis of Baseline SvO_2_, Vasoactive–Inotropic Score, and Oxygen Extraction Ratio for Prediction of 30-Day Mortality

Kaplan–Meier analysis indicated a difference in 30-day survival between the two SvO_2_ groups, with a higher mortality observed in the low SvO_2_ group. The survival curves differed according to the log-rank test (p = 0.008) (Figure 2). In the multivariable Cox model, low SvO_2_ and chronic kidney disease were associated with 30-day mortality. A higher vasoactive–inotropic score was also associated with mortality. In contrast, age and lactate were not significantly associated with mortality in this model.

**Fig. 2.**
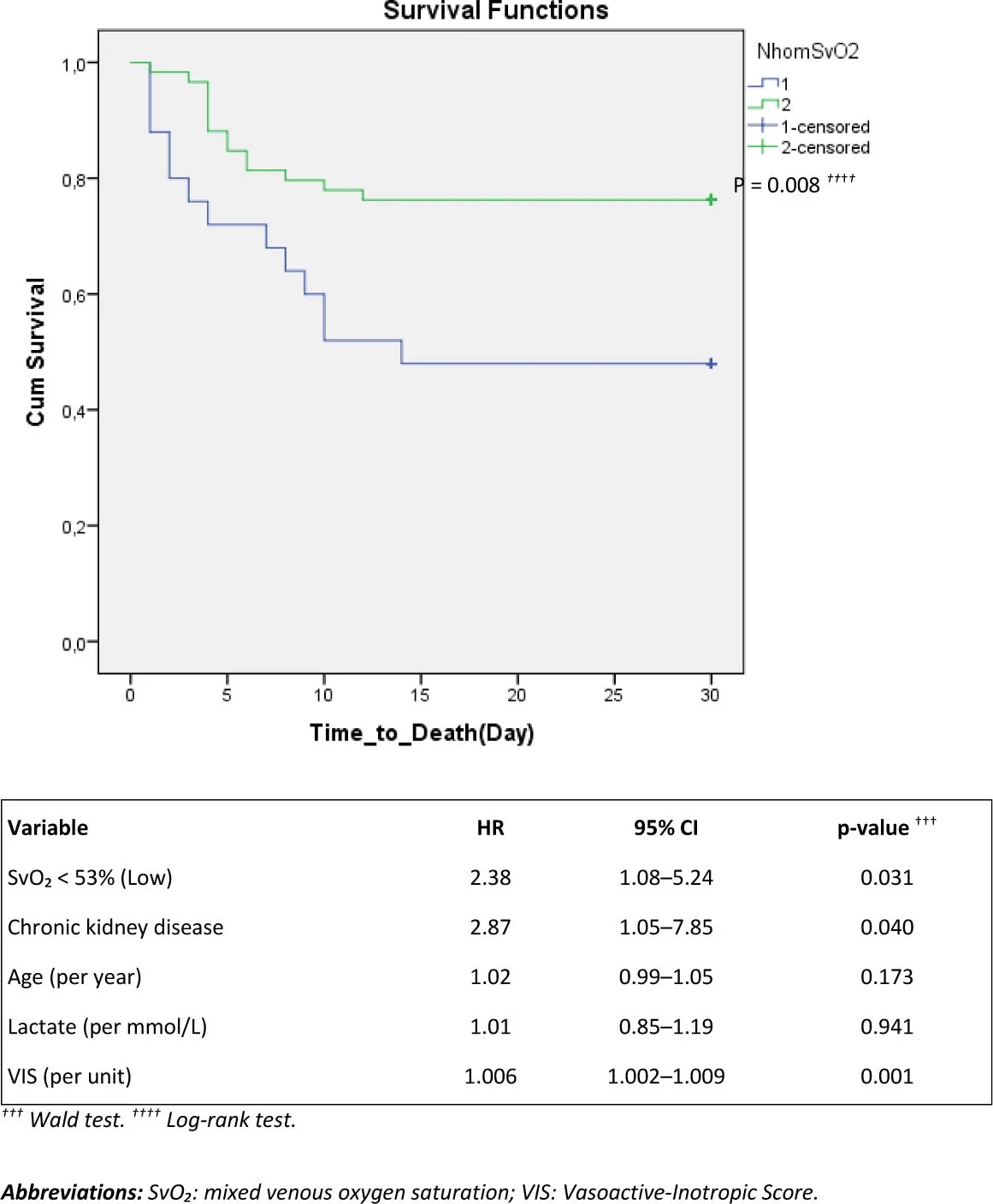
Kaplan–Meier Survival Analysis and Multivariable Cox Regression According to Baseline SvO_2_

Risk stratification according to SCAI stage and SvO_2_ is illustrated in Figure 3. In SCAI stage D, mortality was higher in patients with SvO_2_ <53% than in those with SvO_2_ ≥53% (p = 0.042; RR = 2.43; 95%CI: 1.14–5.18). In SCAI stage E, mortality was also higher in the SvO_2_ <53% group. However, the difference was not statistically significant (p = 0.252; RR = 1.89; 95%CI: 0.73–4.91).

**Fig. 3.**
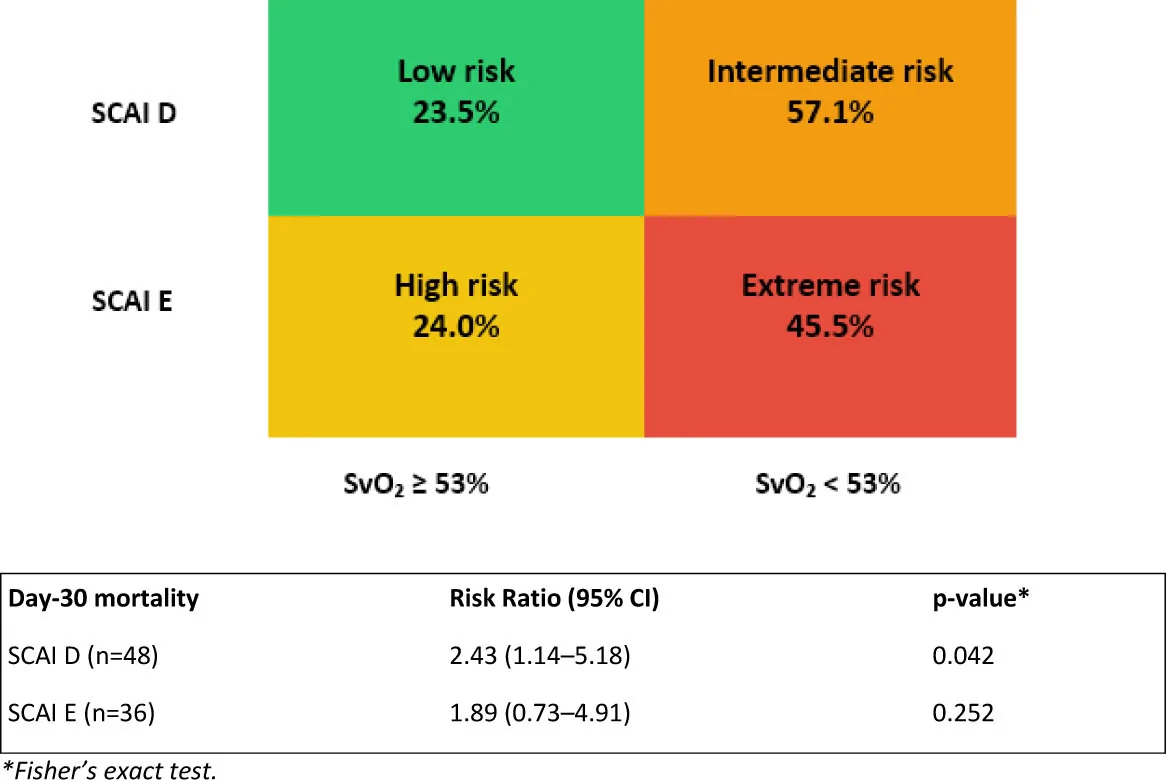
Risk Stratification for 30-Day Mortality by SvO2 categories within SCAI Stages

## Discussions

### Baseline Demographic and Clinical Characteristics of Survivors and Non-Survivors

In our study, left ventricular ejection fraction (EF%) at baseline (T0) did not differ significantly between survivors and non-survivors; EF% was no longer associated with an increased risk of in-hospital mortality [8]. Similarly, hs-troponin T concentrations at time T0 did not show a statistically significant difference between survivors and non-survivors (Table 1). These findings are consistent with the trend reported in a 2022 study by Navin and colleagues, in which both patients with cardiogenic shock due to acute myocardial infarction and those with cardiogenic shock on the background of chronic heart failure demonstrated higher ALT and total bilirubin levels in the in-hospital mortality group (p <0.001) [9]. In cardiogenic shock, phenotypes characterized by right ventricular dysfunction or biventricular dysfunction have been reported to be associated with venous congestion and metabolic derangements, often accompanied by elevated AST, bilirubin, and lactate levels at baseline [10]. Outcomes in cardiogenic shock may depend more on dynamic hemodynamic processes and resuscitation responses, such as changes in organ perfusion, effective circulatory flow, cardiac output, and venous congestion than on baseline static parameters, including EF%, hs-troponin T, or general ICU severity scores [11].

### The Hemodynamic Physiological Narrative of Cardiogenic Shock

Cardiac index did not differ significantly between the two groups (Table 3). Yet cardiac index is a composite variable dependent on both stroke volume (SV) and heart rate (HR); therefore, the absence of a difference in cardiac index does not imply similar hemodynamic states between survivors and non-survivors. This result is not entirely consistent with the study by Navin and colleagues published in 2022, which demonstrated statistically significant differences between survivors and non-survivors when comparing HR, MAP, SBP, and CI [9]. These findings suggest that SVI should be monitored alongside CI throughout the course of treatment to ensure optimal tissue perfusion in patients with cardiogenic shock [12]. In contrast, Cardiac Power Output (CPO) and Cardiac Power Index (CPI) differed between the two groups. This finding is consistent with the study by Popovic and colleagues in a cohort of 85 patients with myocardial infarction (69% presenting with shock), in which multivariable regression analysis demonstrated that the cardiac power index (CPI) at 6 hours was an independent predictor of in-hospital mortality[13,14]. In cardiogenic shock secondary to acute myocardial infarction, reduced cardiac output compromises systemic oxygen delivery, promoting increased peripheral oxygen extraction to meet tissue metabolic demands and thereby lowering SvO_2_ [15,16]. In the present study, the physiological consequences of impaired systemic perfusion were manifested by significantly lower pulmonary artery SvO_2_ in non-survivors compared with survivors. Consistent with this interpretation, Ostadal and colleagues reported that among 95 patients with cardiogenic shock, an SvO_2_ <60% was associated with a 1-year mortality rate as high as 88% [17]. More importantly, SvO_2_ measured via Swan–Ganz catheterization is lower in non-survivors than in survivors and is accompanied by substantially greater vasoactive support requirements, underscoring its relevance as a marker of hemodynamic severity and adverse outcome [18,19].

### Receiver Operating Characteristic Analysis of Baseline SvO_2_, Vasoactive–Inotropic Score, and Oxygen Extraction Ratio for Prediction of 30-Day Mortality

Receiver operating characteristic (ROC) analysis demonstrated that SvO_2_ measured at T0 was associated with 30-day mortality (Figure 1), with an AUC of 0.699. VIS and O_2_ER also showed moderate discriminative ability (AUC 0.660 and 0.650, respectively). Although SvO_2_ showed numerically greater discrimination than VIS and O_2_ER, pairwise AUC comparisons using the De-Long method revealed no statistically significant differences among models (all p > 0.05). The optimal cut-off value for SvO_2_ was 53%. The DanGer Shock Trial conducted by Planka and colleagues incorporated an SvO_2_ threshold <55% into the selection criteria for patients with cardiogenic shock due to ST-segment elevation acute myocardial infarction to guide the use of Impella CP [20]. In the same year, Ostadal and colleagues further highlighted the clinical relevance of SvO_2_ as one of three physiological criteria (alongside cardiac index and Pv–aCO_2_) in the selection of patients for early ECMO. Among patients with cardiogenic shock and SvO_2_ <60%, early ECMO initiation was associated with lower all-cause mortality at 360 days [17]. Pulmonary artery catheterization (Swan–Ganz) uniquely enables direct sampling of true mixed venous blood from the pulmonary artery. Mixed venous oxygen saturation, therefore, reflects the oxygen saturation of blood after complete mixing of venous return from the superior and inferior vena cava within the right heart. As a result, SvO_2_ represents a global indicator of the balance between systemic oxygen delivery and oxygen consumption. In contrast, central venous oxygen saturation (ScvO_2_), obtained from the superior vena cava, reflects only regional venous drainage and may not fully capture whole-body oxygen extraction.

### Combined SCAI Classification and SvO_2_ for 30-Day Mortality Risk Stratification

Within the same SCAI shock stage, SvO_2_ appeared to further stratify patients according to 30-day mortality risk (Figure 3). Among patients with cardiogenic shock secondary to acute myocardial infarction, those with SvO_2_ <53% had higher mortality compared with those with SvO_2_ ≥53%, despite being classified within the same clinical stage. This finding suggests that patients within a given SCAI stage may not be homogeneous in terms of risk, and that differences in SvO_2_ may reflect variation in the severity of circulatory compromise not fully captured by clinical staging alone. In this context, SvO_2_ may provide additional information for risk assessment within each SCAI stage. The combined use of SCAI classification and SvO_2_ may therefore offer a more detailed characterization of patient risk, while remaining consistent with the existing clinical framework for cardiogenic shock.

## Limitations

This study has several limitations. First, it represents a single-center experience, which may limit the generalizability of the findings; the relatively small sample size and limited number of events restricted the number of covariates that could be included in the multivariable Cox regression model. Second, the analysis was restricted to baseline hemodynamic measurements obtained at the standardized time point (T0), immediately after pulmonary artery catheter insertion and before protocol-driven hemodynamic optimization. Therefore, the present report should be interpreted as a characterization of early circulatory physiology rather than an evaluation of treatment effects.

In addition, the prognostic role of SvO_2_ in patients with cardiogenic shock complicating acute myocardial infarction who experienced out-of-hospital cardiac arrest was not specifically evaluated in this study. Further data from this subgroup are required to better define the prognostic implications of SvO_2_ in this clinical setting.

Finally, comprehensive echocardiographic measurements were not systematically available immediately after coronary angiography. Because many patients required urgent stabilization during the early phase of severe cardiogenic shock, complete bedside echocardiographic datasets were not consistently obtained. Consequently, a direct comparison between hemodynamic variables and echocardiographic parameters was not performed.

## Conclusion

In patients with severe cardiogenic shock complicating acute myocardial infarction (SCAI stages D and E), a comprehensive hemodynamic assessment, including cardiac output, cardiac power, and mixed venous oxygen saturation, may provide a more complete characterization of early mortality risk. Our findings suggest that early, standardized assessment in AMI-related cardiogenic shock without baseline mechanical circulatory support may offer additional value for risk stratification within the same SCAI stage. Patients with SvO_2_ <53% were observed to have a higher risk of 30-day mortality.
